# Useful Spectrokinetic Methods for the Investigation of Photochromic and Thermo-Photochromic Spiropyrans

**DOI:** 10.3390/molecules13092260

**Published:** 2008-09-25

**Authors:** Mounir Maafi

**Affiliations:** Leicester School of Pharmacy, De Montfort University, The Gateway, Leicester, LE1 9BH, UK; E-Mail: mmaafi@dmu.ac.uk; Tel.: +44 116 257 7704; Fax: +44 116 257 7287

**Keywords:** Spectrokinetic methods, kinetic elucidation, spiropyrans, photochromic and thermochromic compounds

## Abstract

This review reports on the main results of a set of kinetic elucidation methods developed by our team over the last few years. Formalisms, procedures and examples to solve all possible AB photochromic and thermophotochromic kinetics are presented. Also, discussions of the operating conditions, the continuous irradiation experiment, the spectrokinetic methods testing with numerical integration methods, and the identifiability/distinguishability problems, are included.

## 1. Introduction

**Figure 1:**
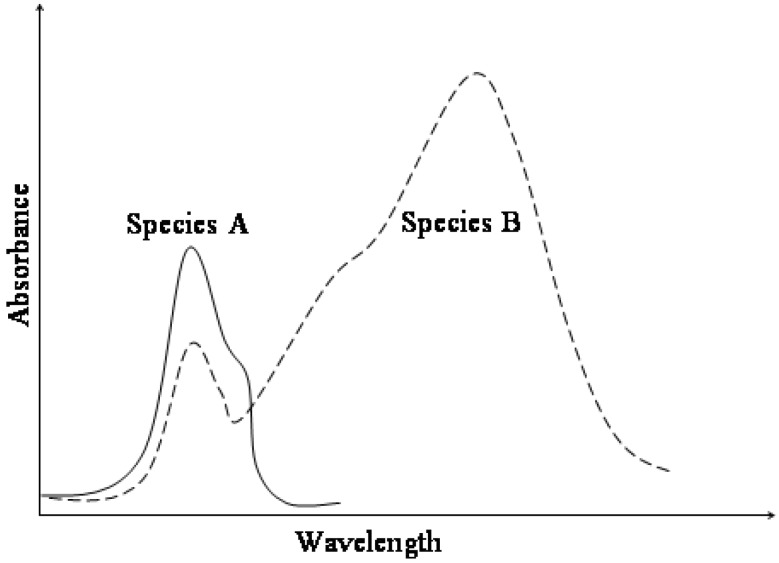
Typical absorption spectra for AB photochromic or thermochromic systems.

Chromism often describes a reversible colour change of a material that can be induced photochemically (photochromism) or thermally (thermochromism) [1,2,3,4]. It translates the transformation of chemical species between two (or more) forms which have different absorption spectra (Figure 1). In the case where only two species can be monitored during the photo- or thermochromism, the reaction is named an AB system (*vide infra* section 2, section 4 and section 5). When both the latter processes responsible for the colour change take place, the AB system is called a thermo-photochromic system.

The initial species A is usually the thermodynamically stable and colourless (or weakly coloured) form whereas the product species B is the coloured form. Although these processes can be observed both in solution and solid state, this review considers only the investigations carried out in solutions.

Organic photochromic, thermochromic and thermo-photochromic compounds of the AB type, like spiropyrans, have known an increasing interest over the last few decades because of their effective and/or potential applications in a number of technologically important fields [1,2,3,4,5,6,7]. Such a position boosted not only the synthetic methods, that has allowed tens of new derivatives to be prepared every year, but also reinforced the need for the development of reliable and easy-to-implement kinetic methods that provide a better insight in the thermophotochromic and photochromic kinetic properties of such compounds [1,2,3,4,5,6,7].

Since the 1960s, continuous research efforts have been conceded for the elaboration of elucidation methods. Dozens of approaches have been proposed thus far for the various AB reaction cases [8,9,10,11,12,13,14,15,16,17,18,19,20,21,22,23,24,25,26,27,28,29,30,31,32,33,34,35,36,37,38,39,40,41,42,43,44]. These methods met with varying success. Most, if not all, were originally constructed on simplifying hypotheses, did not solve the identifiability/distinguishability problems (*vide infra* § 8) and/or required extensive experimental or calculation means. Comprehensive analyses and discussions of these methods and their limitations can be found in several publications [2,3,7,18,45,46,47].

From this perspective, we can identify at least two important interests in developing new and reliable kinetic elucidation methods for AB systems (which should present a better performance and fewer limitations than those already proposed). On one hand, this concern addresses a real need for such efficient methods that do not necessitate coercive experimental conditions and/or extensive data treatments while capable of generating reliable and accurate kinetic and spectroscopic data, i.e., these methods deliver the true kinetic solution. On the other hand, such reliable kinetic results can be put to advantage in the design of new materials endowed with the specific features that are required for particular technological and/or scientific applications.

In order to build up new reliable kinetic investigation tools and useful kinetic elucidation methods, it is imperative that a few general but important requirements are met. For example, the approaches must be based on tight mathematical formalisms, use only observables and avoid as much as possible to rely on assumptions and simplifying hypotheses. They also must clearly identify whether pure kinetic data are sufficient to achieve a complete elucidation (otherwise, the type and the number of supplementary information required for a total elucidation of the kinetics at hand, must be specified). Furthermore, it is necessary that such methods address, if not solve, the identifiability/ distinguishability problems due to the direct incidence of these problems on the reliability of the method itself. Finally, they must be tested against data obtained by an independent technique, such as numerical integration methods (§ 7), before considering their application to experimental data.

Such a strategy has been adopted for the development of the elucidation methods that are reviewed in this paper, which encompass all possible reaction schemes that can govern organic photochromic and thermphotochromic AB systems.

## 2. Physical properties, reactivity and kinetic study of spiropyrans

### Background

**Scheme 1:**
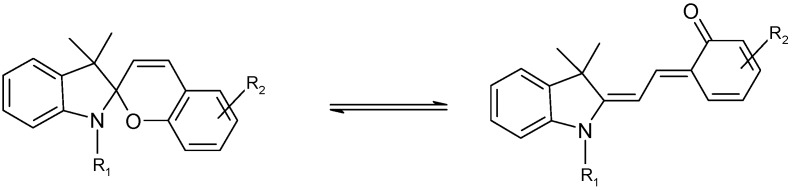
Closed and open forms of spiropyrans.

The chemical structure of spiropyrans involves a benzopyran and an indole moiety joined at a spiro centre (C_spiro_). The two possible enantiomers are separated by an energy barrier of approximately 86 kJ mol^-1^ [48]. Depending on the molecular structure, the reactivity of spiropyran derivatives is believed to be governed by reversible thermal and/or photochemical mechanisms (Scheme 1). Hence, either or both A and B species can be thermally and photochemically reactive and the reaction sequence might include up to two photochemical and two thermal reaction steps.

**Scheme 2:**
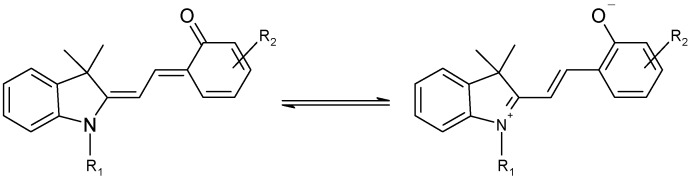
Quiniodal and zwitterionic forms of MC.

The ring opening reaction of spiropyrans (SP) yields a planar merocyanine form (MC) which is characterized by an extended conjugated system (Scheme 1). Both quiniodal and zwitterionic forms can be postulated for MC (Scheme 2). The stability of these resonance forms is sensitive to solvent polarity, with the former likely to be more stable in non-polar media.

The increased conjugation of both resonance forms shifts the electronic absorption of the trans conformer, MC, towards the visible range of the electromagnetic spectrum (Figure 1). Therefore, MC isomers are deeply coloured compared to colourless or light yellow closed-form isomers (SP). This chromism is also accompanied by a significant increase of the ground state dipole moment of the species from 4D for SP to 18D for MC [49].

Both thermal and photochemical transformations of SP into MC are responsible of the breakage of the C_spiro_–O bond. The activation energy of the thermal conversion ranges between 80 and 130 kJ mol^-1^ [46].

**Scheme 3:**
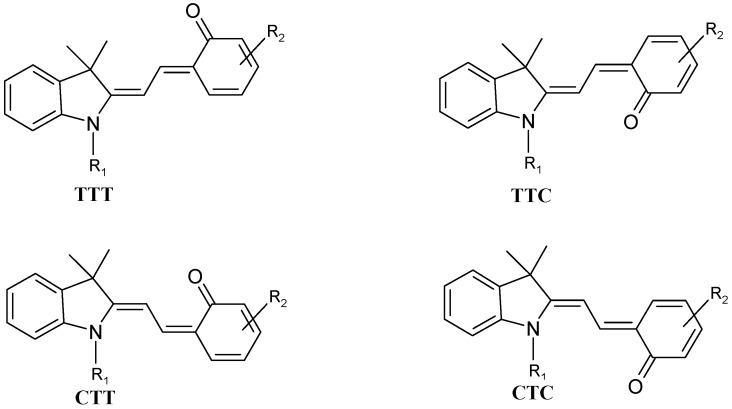
Some intermediates of SP and MC interconversions.

Time resolved studies of the forward SP → MC photoreaction, have demonstrated that the bond cleavage originated from the first excited singlet state of SP (^1^SP*) within less than 200 fs of the excitation pulse [50]. The reaction stages subsequent to the initial bond cleavage have been extensively studied. They ultimately lead to eight possible “*cis*” and “*trans*” isomers of MC. The four *cis* conformations are much less stable than their *trans* counterparts (Scheme 3). Also, according to computational calculations, TTC is the most stable isomer for SP molecules bearing NO_2_ groups [51,52]. Nonetheless, the overall thermal isomerization pathway, the major resonance forms and/or the most stable isomer remain subject to debate.

From a photophysical viewpoint, the fate of the excited state, ^1^SP^*^, produced after light excitation, is dominated by either a singlet or a mixed singlet-triplet pathways depending on whether or not the SP molecule possesses a nitro substituent. The successive transformations leading to the ground state MC (^1^SP^* ^ → ^1^MC_0_), which include cis and trans isomer intermediates (X), have various time scales ranging between tens of picoseconds to a few milliseconds [2,3,50,51,52] (Scheme 4).

The photobleaching process leading to ring closure (i.e. the decay of excited MC, ^1^MC^*^ → SP) has also been investigated by time resolved techniques [53,54]. The phototransformation of MC is believed to proceed via similar types of decay routes (i.e. which can involve a mixed singlet-triplet and/or a singlet pathway depending on whether a nitro substituent is present in the MC molecule). Here as well, the longest lifetime recorded for the intermediates does not exceed a few ms.

The fast photochemical processes observed for both photocolouring and photobleaching of these photochromes has been interpreted by the occurrence of a conical intersection between the ground and excited state potential energy surfaces [55,56].

The thermal recyclisation (MC → SP), on the other hand, obeys first order kinetics in solution. Lifetimes of thermocolouring and thermobleaching reactions were found to be in the sec/min ranges.

**Scheme 4:**
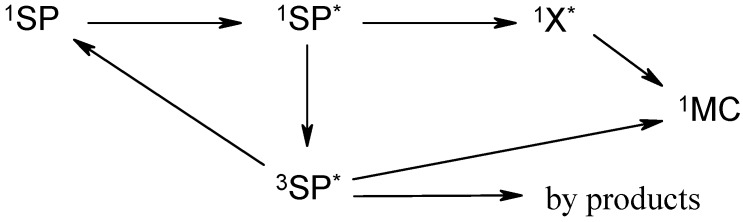
A simplified representation of SP → MC phototransformation.

### Considerations for continuous irradiation experiments

The progress of spiropyran photreactions can also be monitored while the reaction medium is continuously subjected to irradiation. In continuous irradiation experiments, conversely to time resolved studies, the excited state is gradually populated and only long living species can be observed.

The results obtained from time resolved studies clearly showed that amongst the species manifold involved in the photoprocesses of spiropyrans and their merocyanine isomers, only SP and MC (Scheme 1) have lifetimes of over a second. Accordingly, only these two species (and not any of the other intermediates) are expected to be observed and monitored during continuous irradiation experiments (the time resolution of modern diode array spectrophotometers is around 0.5 s). Hence, and even though the real photochemical and thermal mechanisms of such materials are complex, the observed kinetics for these materials can be considered to be similar to that involving bimolecular AB systems. It is however worth noting that in such a situation, the obtained photochemical efficiencies will represent overall rather than unimolecular quantum yields.

There are also a few considerations to be pointed out regarding thermal reactions. Indeed, since both forward and reverse thermal reactions (SP→MC and MC→SP) proceed in sec/min time spans, it is unrealistic to expect separating either of the thermally reactive species (MC and SP) by a physical method. As a matter of fact, the electronic absorption spectra of such transient species are not experimentally accessible and only their kinetic data can be used for elucidation purposes. Therefore, the determination of both species spectra must be considered, for the cases where thermal reactions are involved. This is one of the targets that ought to be met by the kinetic elucidation method (*vide infra* Table 2 and Table 3).

Finally, it should be kept in mind that the irradiation of the initial species with UV light may also initiate a photoreaction of the formed photoproduct (since SP and MC usually share absorption bands in the UV-range). The latter photoreactions’ efficiencies might be irradiation dependent as well. Both these issues cannot be ignored in the absence of tangible information.

## 3. AB systems with similar kinetic behaviour to spiropyrans

**Figure 2:**
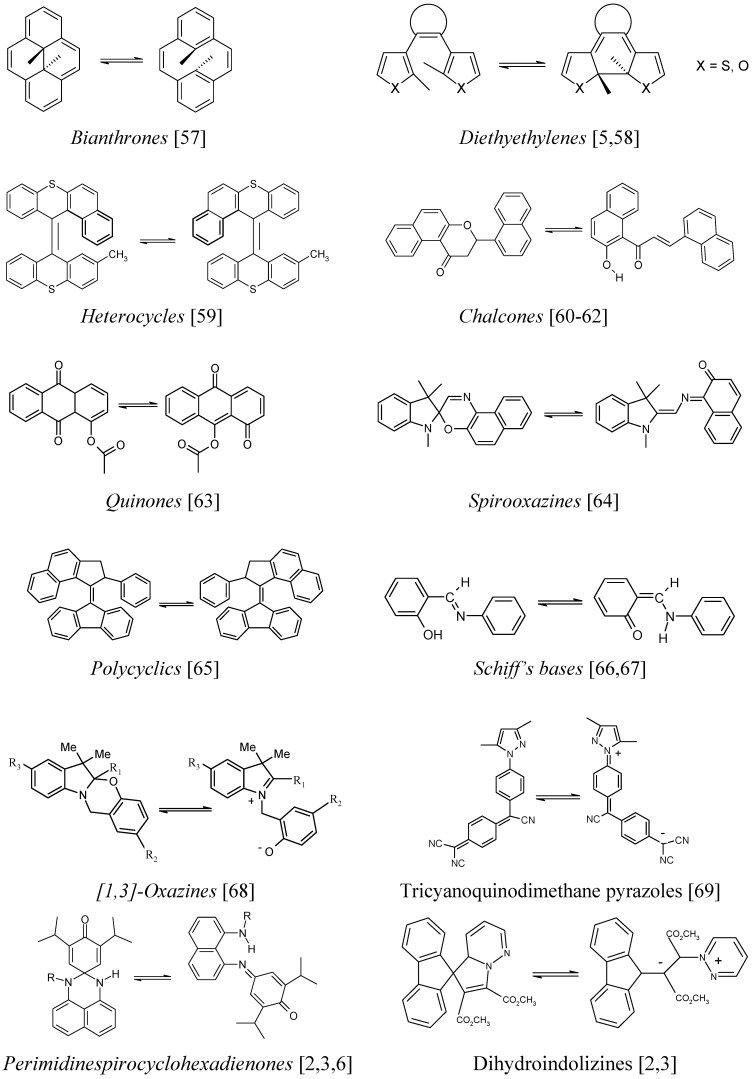
A selection of photochromic and/or thermochromic AB systems possessing a kinetic behaviour similar to that of spiropyrans.

The mechanistic considerations, the kinetic behaviour and the spectroscopic features reviewed above with relation to spiropyran kinetics are also common to a number of families of photochromic and thermo-photochromic materials (Figure 2).

**Figure 3:**
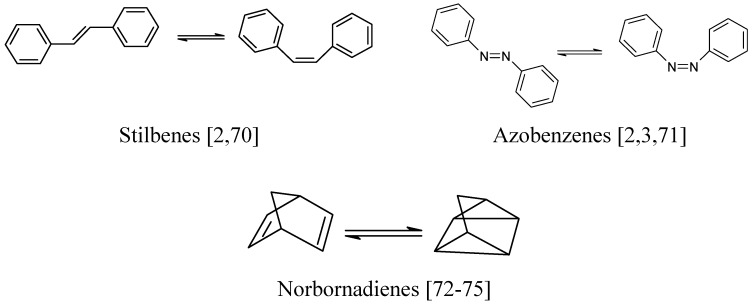
Some non-chromic AB systems.

Hence, the kinetic elucidation methods reviewed below (§ 9) are equally applicable to these materials when their solutions are subjected to continuous irradiation. It is also worth noting that most of these kinetic elucidation methods are useful to investigate non-chromic AB systems as well (Figure 3). 

## 4. Typical kinetic mechanisms

As stipulated earlier in Section 2, the mechanism governing each photochromic and/or thermophotochromic AB system, can encompass up to four reaction steps, of which two are pure photochemical and two are pure thermal processes. Therefore, a total of eleven sequences can be postulated for these reactions (Table 1). Most of these reaction schemes have effectively been postulated for either spiropyrans or other AB systems of similar kinetic behaviour (Figure 2). However, a few less frequent schemes (*S_4_*, *S_8_* and *S_10_*), are included here to complete the series of kinetic elucidation methods for all possible cases of AB reactions.

Photo and thermochromic interconversions might lead to undesirable by products and ultimately to depletion of the active chromic species. Resistance to such degradation (also called fatigue resistance) varies between photochromes of the same series and between families of photochromes. From a mechanistic point of view, this means that at least one reaction step is added to the sequences given in Table 1 (e.g. B→ C with C being the degradation product of species B). Mechanisms including fatigue are not treated in the present review.

**Table 1:** Possible reaction schemes for AB systems.

Reaction	Sequence	Label	References to Spiropyran compounds
A unimolecular photoreaction; AB(1ϕ)	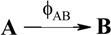	*S_1_*	61,76,77
A unimolecular thermal reaction; AB(1k)	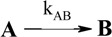	*S_2_*	61
A photochemical reaction reversed by a thermal reaction; AB(1ϕ,1k)	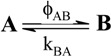	*S_3_*	77, 78
A thermal reaction reversed by a photochemical reaction; AB(1k,1ϕ)	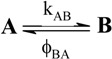	*S_4_*	61,78,79,80
Pure thermal opposed reactions; AB(2k)	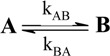	*S_5_*	41,81,82,83
Pure photochemical opposed reactions; AB(2ϕ)	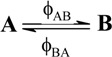	*S_6_*	79,84
Photochemical opposed reactions coupled with a reverse thermal reaction. AB(2ϕ,1k)	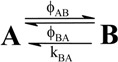	*S_7_*	32,80, 85,86
Photochemical opposed reactions coupled with a forward thermal reaction. AB(2ϕ,1k)	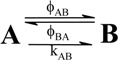	*S_8_*	32,80,85,86
Thermal opposed reactions coupled with a forward photochemical reaction. AB(1ϕ,2k)	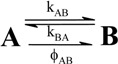	*S_9_*	82,87
Thermal opposed reactions coupled with a reverse photochemical reaction. AB(1ϕ,2k)	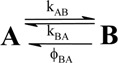	*S_10_*	–
Thermal and photochemical opposed reactions. AB(2k,2ϕ)	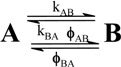	*S_11_*	87,88,89

## 5. Observables and Experimental Conditions

### Photochemical Reactors

The photochemical reactor is a quartz cell where the sample solution is continuously and uniformly stirred and maintained at a constant temperature. The dimensions of the cell may vary (Scheme 4) but usually spectrophotometric cuvettes are used (with a total volume of 1 × 1 × 4 cm^3^). Ususally, the reactor serves also as a sample holder for spectrophotometric measurements during reaction progress.

### Irradiation Conditions

Kinetic investigations using continuous irradiation require that the irradiation (or excitation) beam is monochromatic, of low photon flux and delivered by a highly powered lamp (usually, lasers are not used for this type of experiments). The beam is directed on the sample at an angle to the probing light of the spectrophotometer to avoid interference at the spectrophotometer (or diode array) detector. For this type of experiments, the directions of the probing and irradiation beams are most often perpendicular, i.e. the irradiation is carried out either laterally or top-down ((a) and (b) respectively in Scheme 4). Polychromatic beams are not suitable for continuous irradiation experiments because both the incident light intensity and the photochemical quantum yield values (I_0_ and ϕ, respectively) may vary with wavelength.

**Scheme 4:**
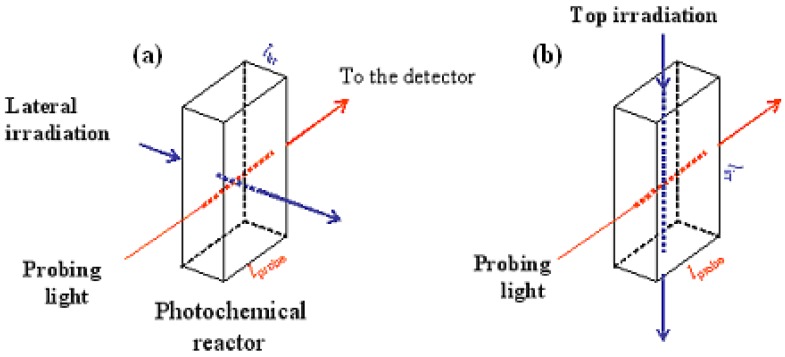
The photoreactor and the possible paths of irradiation and probing lights.

The optical path length of both the probing light (l_probe_) and lateral irradiation beam (l_irr_) correspond to the dimension of the photoreactor (1 cm for the classical spectrophotometric cuvettes). However, for top irradiations (Scheme 4b), l_irr_ will be equal to the length of the liquid solution inside the photochemical reactor that is subjected to the irradiation (e.g. 0 cm < l_irr_ < 4 cm, for a cuvette). In all cases, it is important to identify the (experimental) values of l_probe_ and l_irr_ as they are included in the formalisms of the kinetic elucidation methods (see next section).

The sample can be subjected to two types of irradiations, namely isosbestic and non-isosbetic irradiations. The former corresponds to the situation where the wavelength of the excitation beam (λ_irr_) is identical to that of an isosbestic point (or isosbestic region, see next section) whereas the latter means that λ_irr_ is different from that of an isosbestic point. The elucidation methods developed hereafter may require either or both types of irradiations.

### Observables

Not all fundamental kinetic features and species’ attributes can be measured. The experimentally accessible reaction attributes are called observables. The remaining features are the unknowns. In a typical continuous irradiation experiment, the initial concentration (C_0_), the intensity of the irradiation beam (I_0_), the medium temperature, the optical path lengths l_probe_ and l_irr_ are all measurable quantities which can be defined before the start of the experiment. The accessible kinetic data collected on a given photochromic and/or thermochromic reactive system are represented by plots of the measurable variation of the absorbance (M) with reaction time (t). It is important to underline that spectrophotometric techniques of analysis allow only one absorbance (labelled M), monitored by the probing light at the condition of observation (i.e. where the optical path length is l_probe_), to be recorded. The absorbance of the medium in the condition of irradiation (along the optical path length l_irr_) is usually not directly accessible (Scheme 4), and hence it is not considered to be an observable.

A particular recorded M(t) curve is specific to an irradiation (λ_irr_) and an observation (λ_obs_) wavelength as well as to l_probe_, l_irr_, I_0_ and the medium temperature (T). Such curves are called kinetic traces. They are obtained either during the irradiation or the thermal relaxation of the reactive medium. The photochemical traces relate to the progress of both photochemical and thermal reactions of a given sequence while thermal curves are exclusively due to thermal processes (these traces are recorded in the dark, i.e. I_0 _= 0). Also, it is worth noting that traces for individual species are most often accessible if the reaction medium is observed at a wavelength (λ_obs_) where only one species absorbs (most likely in the visible region of the spectrum because the spectra of reactant and product often overlap throughout the UV region).

A number of other observables may be obtained by alternative (spectroscopic and/or physico-chemical) methods. However, the methods presented hereafter have been built on the hypothesis that kinetic data and the observables listed above are the only output of the experiment. This is because alternative techniques and physical methods are time consuming, expensive and more importantly they are difficult to set up for a majority of sequences (Table 1) due to the transient character of one or both species involved in the reaction. Reference to the use of alternative techniques will be made where appropriate in the following sections.

As alluded to above, in order to perform isosbestic irradiations it is necessary to observe an isosbestic point on the absorption spectra of the reactive medium during its evolution with irradiation time. Thus far, the presence of an isosbestic point was confirmed for all known photochromes and thermo-photochromes. Incidentally, this feature is often used to indicate the smooth progress of the reaction and the absence of by-products. Alternatively, similar irradiations can be carried out using wavelengths situated in isosbestic regions where the spectra of A and B are the same (where the species’ molar extinction coefficients are equal, ε_A_ = ε_B_, over a spectral range).

According to the properties of this type of AB reactions, the photostationary state (PSS) and/or the state of thermal equilibrium (STE) are reached within minutes. They correspond to plateau regions on the kinetic traces recorded at long reaction times. Some of the kinetic elucidation methods reported here require data relative to PSS and STE, and hence these states are considered to be observables.

### The unknowns

The fundamental features that are not directly accessible from the continuous irradiation experiment (the unknowns) are of two types, kinetic and spectroscopic quantities. The unknown kinetic reaction attributes are the quantum yields of the photochemical steps (ϕ_AB_ or ϕ_BA_) and the rate constants of the thermal reactions (k_AB_ and k_BA_). The inaccessible spectroscopic data are the molar extinction coefficients of species A and B (ε_A_ and ε_B_ respectively). Quantum yields and molar extinction coefficients are wavelength dependent while rate constants are temperature dependent. Of course, the total number of unknowns varies with the sequence as indicated in Table 2. In this respect, it is relevant to consider that ε_A_ is unknown for all thermally reactive initial species (A) because the progress of such reactions is immediate following the preparation of the stock solutions. (Reliable spectrophotometric measurements cannot be carried out promptly enough due to relatively fast rate constants, k_AB_ [2,3]). In addition to the above unknowns, the species equilibrium concentrations (at the PSS, C_i_(PSS), and at the STE, C_i_(STE); with i = A or B) are not measurable in the type of experimental conditions previously set and therefore they must be accurately defined by the methods as well.

**Table 2:** Sets of unknowns specific to each kinetic case of Table 1.

Reaction scheme	Possible unknowns for the sequence
*S_1_*	ϕ_AB_ and ε_B_
*S_2_*	k_AB_, ε_A_ and ε_B_
*S_3_*	ϕ_AB_, k_BA_ and ε_B_
*S_4_*	k_AB_, ϕ_BA_, ε_A_ and ε_B_
*S_5_*	k_AB_, k_BA_, ε_A_ and ε_B_
*S_6_*	ϕ_AB_, ϕ_BA_ and ε_B_
*S_7_*	ϕ_AB_, ϕ_BA_, k_BA_ and ε_B_
*S_8_*	ϕ_AB_, ϕ_BA_, k_AB_ and ε_B_
*S_9_*	k_AB_, k_BA_, ϕ_AB_, ε_A_ and ε_B_
*S_10_*	k_AB_, k_BA_, ϕ_BA_, ε_A_ and ε_B_
*S_11_*	k_AB_, k_BA_, ϕ_AB_, ϕ_BA_, ε_A_ and ε_B_

The kinetic solution sought by each elucidation method is represented by the true set of absolute values determined by the method for the unknowns corresponding to the kinetic case considered (see Section 8 and Section 9)

## 6. Fundamental Kinetic Laws

The mathematical formalism required for the build up of the elucidation methods is considered in this section. The labelling employed here (such as a_4_, a_5_, a_19_, M, m_0_….) is similar to that used in the original papers [90,91,92,93,94,95]. A glossary of the symbols and labels adopted in this review is included in Section 13.

In the following, irr, isos and obs stand respectively, for the irradiation, isosbestic and observation wavelengths (where irr ≠ isos, and, obs may or may not be equal to irr). The absorbance (M) and the initial reaction velocity (m_0_) are referred to an excitation and an observation wavelength (λ_exc_/λ_obs_), i.e. either irr or isos for the excitation of the photochemical reactions, and either obs, irr or an arbitrary λ, for the monitoring wavelength. The photokinetic factor (F) which is always defined by the irradiation features (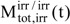, 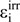, l_irr_...etc) can be time dependent F^irr^(t) or constant when measured at t = 0, F^irr^(t) = 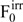, or corresponding to either the PSS, F^irr^(PSS) = 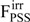, or the STE, F^irr^(STE) = 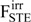. The absorbance 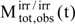 monitored in the observation conditions (l_probe_), is labelled 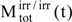 throughout.

### The Fundamental Kinetic Equations for AB Systems

The continuous irradiation experiments must be carried out in a photoreactor where a homogeneous sample is continuously stirred and irradiated at a constant temperature. The fundamental differential equation describing the variation of species concentrations with reaction time (Eq.1) is set for the case where the wavelength of the monochromatic irradiation beam (λ_irr_) is supposed to correspond to a spectral region where species A and B absorb different amounts of the incident light, and the concentration of the excited state remains negligible. C_A_(t) and C_B_(t) are the concentrations of species A and B, respectively. Eq.1 corresponds to the most inclusive reaction mechanism *S_11_* (in Table 1). Hence, individual differential equations for the remaining *S_1_*-*S_10_* sub-sequences can easily be inferred from Eq. 1.

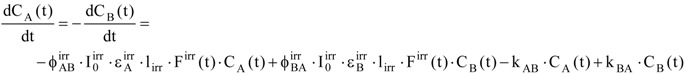
(1)
The F^irr^ is the variable photokinetic factor, which is expressed as a function of the total absorbance at the irradiation wavelength (l_irr_),

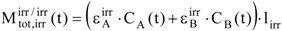
(2)
and which formula is

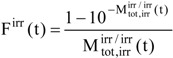
(3a)

Constant values of the photokinetic factor can be calculated for various situations including for the initial absorbance recorded at the start of a photochemical reaction (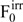), the absorbance reached at PSS (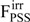) or STE (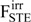), and the isosbestic absorbance (F^isos^). These photokinetic factors are given by the following equation where only observables are used (with, in Eq. 3b, init = 0, STE or PSS),

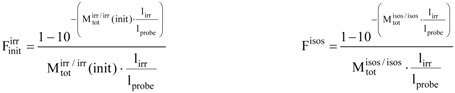
(3b, c)

The fundamental equation (Eq. 1) can be rearranged to express the rate of change of the total observed absorbance

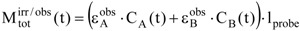
(4)
where the sample is irradiated at λ_irr_ and observed at λ_obs_ (with λ_irr_ ≠ λ_obs _), as

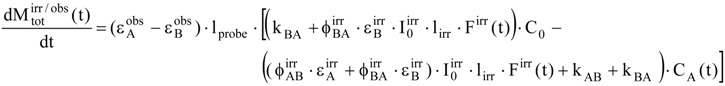
(5)
From Eq. 5 we can derive the general expression of the initial velocity (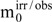) of the reaction, as

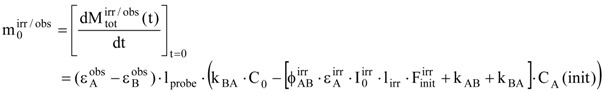
(6)
where, depending on the reaction considered, the argument “init” can be equal to 0, PSS or STE.

The mass balance equation gives the relationship between the species concentrations at time t, the initial time (t = 0), STE and PSS and the total concentration C_0_, as

C_0_ = C_A_(t) + C_B_(t) = C_A_(0) + C_B_(0) = C_A_(STE) + C_B_(STE) = C_A_(PSS) + C_B_(PSS) (7)

It is also possible to extract the individual analytical expressions of the species concentrations at PSS and STE (at these states, the absolute value of the concentration is considered invariant with reaction time, hence, dC_A_/dt = 0).

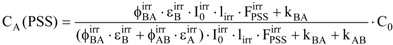
(8.a)

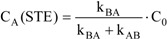
(8.b)
and hence C_B_(PSS) and C_B_(STE) can be worked out from Eqs. 7 and 8. By using the latter equations in the equation of 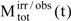, Eq. 4, we can write 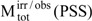 formula, as

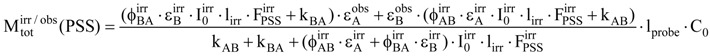
(9)

From a mathematical viewpoint, Eqs. 1 and 5 cannot be solved through a closed-form integration. The difficulty is due to the time dependence of the photokinetic factor (F as given in Eq. 3a). The only known exception is that of the unimolecular photoreaction AB(1ϕ) whose differential equation can be analytically integrated [93]. The corresponding kinetic model is reviewed at the beginning of Section 9.

However, the differential equations (Eqs. 1 and 5) are readily solved for thermal reactions and also for photoreactions if F remains constant. This condition is met whenever the wavelength of the monochromatic irradiation beam is equal to that of an isosbestic point (§ 6). The general equations relative to these cases are presented in the next section.

### Basic Kinetic Laws for AB Kinetics When F is Constant

The kinetic laws, representing the temporal variation of the species concentrations (C_A_ and C_B_), for all AB(nk,nϕ) systems studied under isosbestic irradiations (with 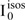 its intensity), whether they involve pure photochemical, pure thermal or a combination of photochemical and thermal reaction steps, have been defined [90] as:

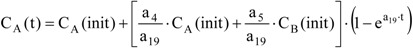
(10a)

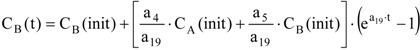
(10b)
where init = 0, STE or PSS, as previously set. The a_i_ coefficients are functions of the kinetic parameters [90]; they are defined for the photochemical reaction (where both the thermal and the photochemical processes operate, and indexed by the letter P, a_iP_) and the thermal reaction (indexed T, a_iT_, where only the thermal processes are active, i.e. after the irradiation is cut off and 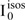 = 0). Hence, for an AB(2k,2ϕ) system as given by Sequence *S_11_* (Table 2), we obtain :

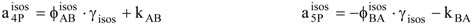
(11a,b)

a_4T_ = k_AB_   a_5T_ = −k_AB_ (12a,b)

Coefficient γ_isos_ in Eqs. 11 depends on the attributes of the isosbestic point (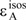,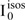,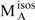), the irradiation condition (l_irr _= l_isos_) and F^isos^, the photokinetic factor for the isosbestic irradiation (Eq. 3b).

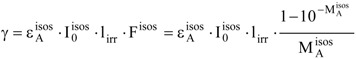
(13)

The overall observed rate constants of the photochemical and the thermal reactions are related to the exponential factors in Eqs. 10, as follows [90]

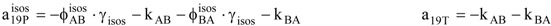
(14a,b)

The cumulative absorbances (M) of the species A and B at an arbitrary observation wavelength considered at init = 0, PSS and STE, are respectively defined by

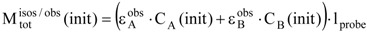
(15)

Similarly, the cumulative initial velocities of the various thermal (Eq. 16a) and photochemical (Eq. 16b) reactions are given by

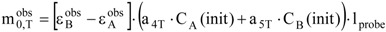
(16a)

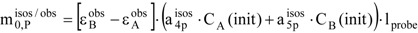
(16b)

The equilibrium concentrations of the species (that might be used in Eqs. 10, 15 and 16) are defined on the basis of the respective kinetic coefficients a_i_ (Eqs. 11 and 12) and the total initial concentration.

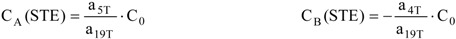
(17a,b)

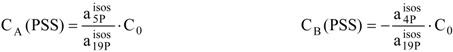
(17c.d)

On the basis of Eqs. 11-17, we can rearrange Eqs. 10 so that a general formula (Eq. 18) is set for the time dependence of the cumulative absorbances (i.e., where the two species are simultaneously monitored at the observation wavelength; with symbols Ω = P or T, and θ = isos for photoreactions).

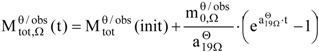
(18)

Eqs. 10-18 can be used for any sequence in Table 2 provided that the missing parameters and coefficients in each kinetic reaction are given the zero value. They also represent the model equations employed to fitting simulated and experimental traces.

## 7. Data Simulation, Fitting of the Kinetic Traces and Testing the Methods

### Data simulation

Numerical integration methods (NIM) are important tools to calculate the values of integrals, which are specially crucial for non-solvable differential equations (e.g. Eqs. 1 and 5). Hence, kinetic traces for any system amongst those listed in Table 2 can readily be constructed by feeding the numerical integration of Eq. 1 with chosen realistic and physically meaningful values of the involved parameters. Such calculations may include cases of constant and variable photokinetic factors as well as photoreactions for which quantum yields are wavelength dependent. The simulated data presented hereafter were obtained with a fifth-order Runge-Kutta numerical integration method within Mathcad premium software (version 2001i).

### Fitting of the Kinetic Traces

The data treatment for such kinetics starts with fitting the traces with the appropriate model equations (Eqs. 18). Each simulated and/or experimental trace is fitted and the corresponding fitting coefficients for the model-equation are extracted. These are 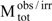
(0 or PSS), 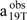 and 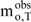 for thermal reactions, and, 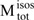
(0 or STE), 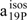
and 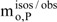 for photoreactions. The kinetic curves may be increasing or decreasing. The fitting coefficients are used further to carry out the elucidation method corresponding to the considered kinetic system. 

A number of available software programmes can readily perform such non-linear curve fittings. In this paper a Levenberg-Marquardt iterative programme within the Origin 6.0 software package were used for the determination of the best fit curves and the extraction of the fitting coefficients.

### Testing the Methods

Prior to applying the methods to experimental data, it is necessary to confirm their validity. One way to achieve this confirmation would be to test the methods with data generated by a completely independent mathematical approach, i.e. the numerical integration methods (see *data simulation* section above). It is a reasonable strategy because the mathematically analytical elucidation methods should stand against any independently generated data like those provided by NIMs. The advantage here is the perfect knowledge of all reaction attributes. Indeed, the method is accepted as valid if it is capable to retrieve those values of the attributes originally used for NIM.

Furthermore, as NIMs afford the possibility of simulating a great number of reaction cases and experimental conditions, they can represent an efficient tool for testing the elucidation methods in a large set of possible situations. This has advantage over the limited number of data afforded by experiments on a particular reaction case. Nonetheless, after the methods have been positively tested against NIM data, they should be applied, in a subsequent stage, to particular experimental results. 

The testing procedure is as follows: let *set A* be the set of parameter values (ϕ, k and ε) that has been used to feed the numerical integration of Eq. 1 and led to the generation of photochemical and thermal kinetic traces (e.g. Tr_P_ and Tr_T_, respectively). Then, in order to achieve the conditions of a realistic experiment, Tr_P_ and Tr_T_ together with C_0_, I_0_, l_probe_ and l_irr_ are considered as observables of known values whereas *set A* will represent the experimentally inaccessible set of unknowns that the method must define (i.e. set A, including the values of ϕ, k and ε, will remain unknown during the rest of the treatment). If data are obtained by an isosbestic irradiation, the traces Tr_P_ and Tr_T_ are fitted to the appropriate model equations and their respective fitting coefficients determined. In the case of non-isosbestic irradiation, the equilibrium absorbances and/or the initial velocities are determined. The adequate spectrokinetic method is then applied and an elucidation solution is found. The latter elucidation solution, corresponding to a set of values of the unknown parameters, is called *set B*.

Since the spectrokinetic methods have been built from mathematically analytical formalisms, *sets A* and *B* should incorporate the same value for each parameter. A high degree of precision should characterize these values (theoretically they should be exactly identical). Otherwise, any discrepancy between the values of *sets A* and *B* would strongly indicate that the spectrokinetic method at hand is flawed and therefore cannot be considered as an elucidation method for real kinetic cases.

It is worth noting that this strategy differs from that using the experimental data and traces as a control set while the parameter values feeding the RK integration are changed until experimental and calculated data show a good agreement. The difference lies in the fact that the “good agreement” found in such a way may only represent a convergence to a local minimum or to one of several identical minima. Therefore, this classical approach does not necessarily lead to a unique kinetic solution, but more importantly, it does not generate the true solution as an exclusive output (several solutions are found if several identical minima are possible). This issue is developed further in Section 8.

## 8. Distinguishability and Identifiability Problems

The purpose of a kinetic elucidation method is to determine with certainty the reaction model and the values of the unknown kinetic and spectroscopic parameters defining the dynamics. This means that the only successful elucidation methods are those able to properly address and lift any ambiguity relating to the uniqueness of both the specific attributes of a kinetic problem and the mechanism governing its reaction [96,97,98,99,100].

Typically, ambiguity occurs when different sets of parameters’ values and/or reaction models lead to an equal goodness of fit between the experimental data and the chosen kinetic model. The root cause for such a situation is the excess number of unknown parameters compared to the number of the linearly independent equations describing the kinetics. The problem is even more acute if the latter equations are non-linear (even though they are linearly independent). As a consequence, the experimental data collected on a reaction dynamics, and which is used for the purpose of kinetic elucidation, can be interpreted in different ways, i.e. leading to a number of kinetic solutions sharing a similar probability of occurrence. The total number of such solutions can exceed the number of equations or even be infinite. This set of kinetic solutions represents what is called *a degenerate solution* [98,100].

The degeneracy of the solution can be seen through what is generally known as the distinguishability and identifiability problems.

In the cases where more than one reaction scheme reproduces the experimental data with equal goodness of fit, the system is said to be non-distinguishable. For example, kinetics relative to schemes *S_3_* and *S_7_* which involve respectively two and three reaction steps, are non-distinguishable from one another if not solved analytically. Indeed, both are represented by the same model Eqs.18, i.e. their experimental traces can both be fitted by the same Eqs. 18 and in both cases the corresponding fitting coefficients are extracted (§ 7). Therefore, at such a simple analysis level (i.e. if the kinetic analysis is only limited to observing a good fit of the traces with Eqs. 18), the distinguishability problem will remain undoubtedly unsolved. The requirement for more powerful elucidation methods is then required.

The identifiability problem is raised with respect to a given reaction scheme for which more than one set of values for the unknown parameters, enables the kinetic model to fit the experimental data with high accuracy. The kinetics of *S_11_* is an obvious example for identifiability when only kinetic data is used (§ 9).

Therefore, a successful elucidation method is not the one that achieves a unique solution (i.e. any option amongst the set of the degenerate solution), but is the one capable of finding the kinetic option that stands for the true solution. Hence, solving the identifiability/distinguishability problems is a central issue for such kinetic studies.

In this respect, the elaboration of elucidation methods that allow singling out the true solution is fundamental for studying the kinetics of AB systems.

For most spiropyrans kinetics and similar AB systems (Table 2), the question is whether fully efficient kinetic methods are achievable? The fulfilment of this goal will require that the elucidation procedures are built upon assumption free strategies, and employing tight formalisms. The methods presented in the next section, have been developed with these requirements in mind.

The testing strategy based on NIM simulations (§ 7) represents a good way of tackling identifiability and distinguidshability problems. When the true solution of a kinetic system is unequivocally met with such tests, it means that the identifiability problem is solved and the reliability of the method employed is proven.

## 9. The Spectrokinetic Elucidation Methods

The spectrokinetic methods presented below have been obtained through tight mathematical analyses, based on the equations of Section 6. This set of equations is sufficient to reach analytical kinetic solutions for a majority of the reaction cases revealed in Table 1 and which involve, for each reaction, the maximum number of possible unknowns (Table 2). For the purpose of clarity, only the main results will be reported here. The detailed formalisms of the elucidation methods can be found in the original papers [92,93,94,95]. The variability recorded on the number of both unknowns and accessible kinetic traces for the various reactions, makes that each reaction sequence requires a particular elucidation method, as has been the case in the literature. So far, a unique elucidation method capable of dealing with all kinetic cases and solving their identifiability/ distinguishability problems, is yet to be invented. 

### AB(1ϕ) reactions, S_1_

For the unimolecular photoreaction, the progress of the reaction is induced by the irradiation of the sample with a light that selectively hits species A (and not B), i.e. for this reaction case a non-isosbestic irradiation is necessary.

As pointed out above (§ 6), the fundamental differential equation Eq. 5 has been solved for *S_1_*-type reactions [93]. The closed-form integration of Eq. 5 is achieved with a variable separation method after proceeding with a change of variables. The irradiation and observation wavelengths (λ_irr_ = λ_obs_) must be equal. The resulting kinetic “Log-exp” model is [93]:

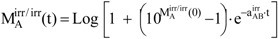
(19a)
where, Log is the base 10 logarithm and the coefficient 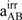 represents the wavelength-dependent overall photoreaction rate, defined as

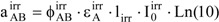
(19b)
with Ln the neperian logarithm. The parameter 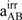 is the fitting coefficient of Eq. 19a. The photoreaction efficiency, 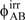, is the unknown and all the remaining quantities in Eqs. 19 are experimentally accessible.

**Figure 4:**
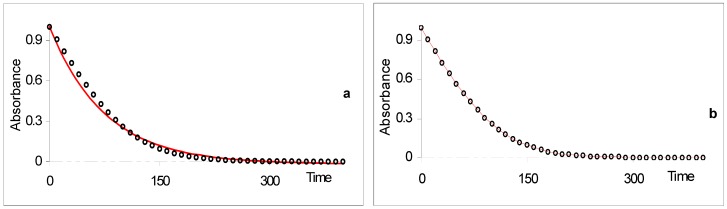
Comparison between fitting simulated RK data to (a) monoexponential and (b) analytical model Eq.19a [reprinted from Ref. 93 with permission of the Journal of Photochemistry and Photobiology: A Chemistry].

A good agreement characterises the results of the model when tested against simulated RK data. It fits well the simulated kinetic traces for the disappearance of the reactant (A) while the same set of RK data is poorly reproduced by a monoexponential model (Figure 4).

**Scheme 5:**
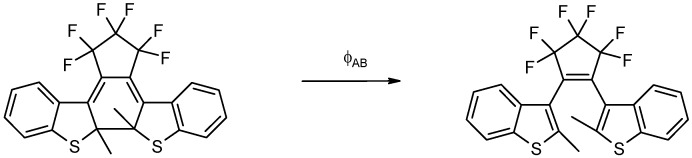
Unimolecular phototransformation of the closed into the open form of a diarylethene.

**Figure 5:**
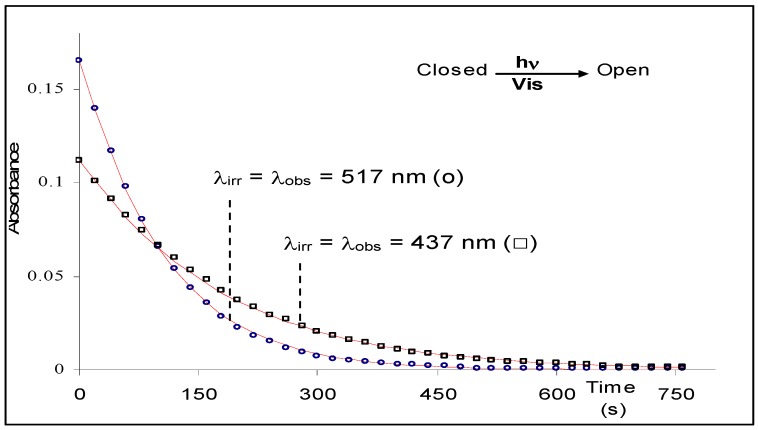
Photokinetic traces in hexane solution (1.82 10^-5^ M (ο) and 3.28 10^-5^ M (□); 15°C) at two irradiation wavelengths in the visible. Experimental data (circles and squares) are readily fitted by the theoretical model, Eq.19a (lines) [reprinted from Ref. 93 with permission of the Journal of Photochemistry and Photobiology: A Chemistry].

Also, Eq. 19a has been successfully applied to the kinetic transformation of the open form of a photochromic diarylethene derivative into its closed form using visible light (Scheme 5). Both isomers are thermally stable. Photoirradiations at two wavelengths, 437 and 517 nm, were performed separately on the open isomer. The kinetic traces for both processes were fitted with Eq. 19a. The good agreement between the experimental data and the model is shown in Figure 5 [93].

The determination of the fitting parameters (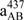 and 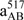) has allowed the determination of the absolute value of the quantum yield (i.e. the unknown system) at each irradiation wavelength, 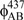 = 0.28 and 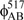 =0.24.

The model is believed to be useful to the classical example of the photoreduction of potassium ferrioxalate, whose kinetics is considered to occur via a single photochemical step (Fe^3+^―h*v*→ Fe^2+^). So far, the experimental data of the ferrioxalate actinometery were usually analysed by Runge-Kutta or Simpson’s numerical integration methods [2,3].

### AB(1k) Systems, S_2_

This is a classical first order thermal reaction which is fully described by a monoexponential model Eq. 20 (extracted from Eq. 18). It is a typical AB reaction that involves a single thermally active species (either A or B). The reaction corresponds to the thermal relaxation of one isomer in the dark, which ends when the thermally stable species had been fully produced in the reaction medium.

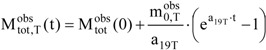
(20a)

Both increasing and decreasing traces are readily fitted by the model equation (Eqn. 20a). Since the exponential coefficient a_19T_, which is either equal to (– k_AB_) for A→B transformations or (– k_BA_) for B→A reactions (Eq. 14b), is easily obtained from the curve fitting, the determination of the reaction rate constant is straightforward.

**Figure 6:**
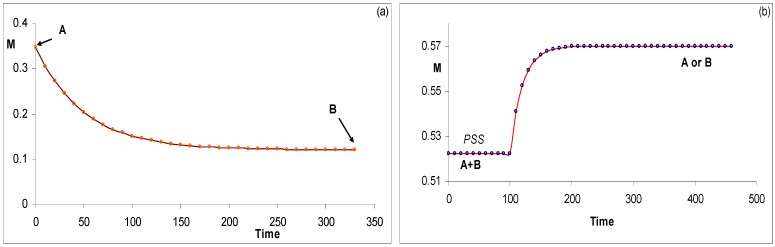
Examples of unimolecular thermal reaction traces.

(a) The reaction starts from an initial concentration of the reactive species, and (b) it starts from a state where A and B are already in a photoequilibrium.

In realistic situations however, the recording of the early stages of the reaction is difficult to achieve in solution if the initial thermochromic material (A) is the species monitored (because most often short lifetimes characterise such thermochromic interconversions).

In the cases of sequences *S_3_*, *S_7_* and *S_8_* (Table 2), the thermal relaxation of the species starts from the PSS (Figure 6b) and hence a similar formula to Eq. 20a is used to fit the data but where the initial absorbance is replaced by that observed at the PSS (Eq. 20b and Figure 6b).

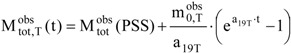
(20b)

### AB(1ϕ,1k) Systems, S_3_

**Figure 7:**
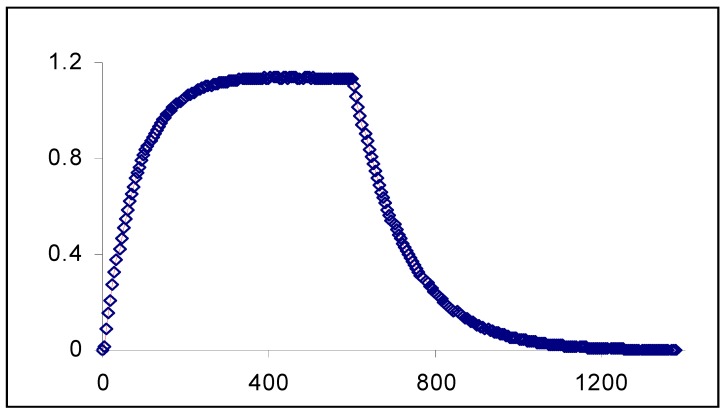
Kinetic traces of the photocolouring and the thermal fading reactions of NO_2_-BIPS (Scheme 6) in ethyl acetate (plots represent Absorbance *vs.* Time (in s)). The experimental data (squares) are fitted by model Eqs. 21a and 20b (solid lines). T = 14˚C, C_0_ = 5.83 10^-5^ M, λ_isos_ = 339 nm, λ_obs_ = 580 nm [reprinted from Ref. 92 with permission of the International Journal of Chemical Kinetics].

This reaction involves a phototransformation reversed by a thermal relaxation (which are characterized by a quantum yield ϕ_AB_ and a rate constant k_BA_, respectively). The reaction progress, under irradiation, leads to a PSS from which the thermal relaxation takes place in the dark (Figure 7). When an isosbestic irradiation is used, the model equation for the photochemical reaction is [92]

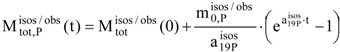
(21a)

The fitting of the photochemical and thermal traces to Eqs. 21a and 20b, respectively, yield the following fitting parameters: 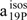, 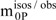, 
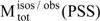
, a_19T_ and 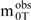.

**Scheme 6:**
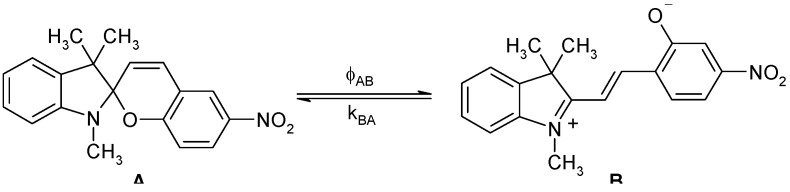
Reaction of spiropyran NO_2_-BIPS.

The elucidation of this kinetics can be achieved by using only pure kinetic data. The true solution (consisting of the absolute values for the three unknowns, Table 2) as well as the full spectrum of transient species B can be worked out as follows.

The value of the thermal reaction rate constant is obtained from the fitting coefficient a_19T_ (which is equal to (– k_BA_) as described for data treatment corresponding to *S_2_* kinetics), and the absolute value of the quantum yield is obtained as

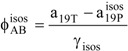
(21b)

Once these two parameters (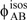 and k_BA_) are known, the calculation of species concentrations at PSS is achieved on the basis of Eqs. 17c and 17d. This allows the determination of the exact value of the molar extinction coefficient of the transient species (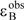 at the observation wavelength) from the equation and value of 
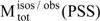
 (Eq. 15, with init = PSS). Accordingly, the kinetic elucidation is completed.

**Figure 8:**
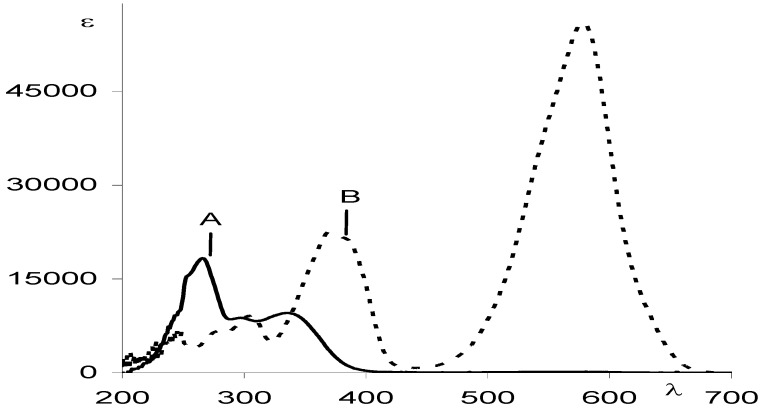
Absorption spectra of species A (plain line) and B (dashed line) for Scheme 6. The spectrum of B has been reconstructed using data from the elucidation method and Eq.21c. (ε in L.mol^-1^.cm^-1^ and λ in nm) [reprinted from Ref. 92 with permission of the International Journal of Chemical Kinetics].

The reconstruction of the spectrum of species B is achieved by applying either terms of Eqs. 21c to different observation wavelengths throughout the absorption region (Figure 8). (λ is an arbitrary wavelength that takes any value where A and B absorb).

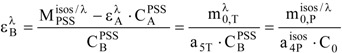
(21c)

### AB(1k,1ϕ) Systems, S_4_

The elucidation of this kinetic case is much simpler than that of *S_3_* because species B can be obtained in a stable form and hence its spectra can be recorded. When species B is the coloured form [78], species A can be regenerated by applying a non-isosbestic irradiation to B and the efficiency of the photoreaction at a given irradiation wavelength (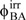) is determined as per *S_1_*. Once the phototransformation of B is complete (i.e. 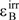) and while keeping the photoirradiation light on, the full spectrum of species A can be recorded. The thermal rate constant k_AB_ is subsequently obtained by fitting the thermal relaxation trace of species A (recorded in the dark) to the model Eq. 20a (the same treatment used for *S_2_* applies). The photochemical efficiencies at wavelengths where both species absorb can be derived from Eq. 6 (where 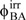 = k_BA_ = 0).

In the case where species B is the colourless form, the thermal transformation of species A into B will generate a similar situation to that described for *S_3_* (i.e. the elucidation method developed for the kinetic case *S_3_* applies except that the labelling of the equations must take into account that now the initial species is B).

The elucidation of this kinetic case is simpler than the preceding one (*S_3_*) because the full spectra of both species are easily accessible.

### AB(2k) Systems, S_5_

The systems involving pure and opposed thermal reactions cannot be solved on the basis of kinetic data alone if the early stages of the starting reaction are not accessible (*S_2_* kinetics and Table 2). Alternative physical data can be considered. In this instance, the determination of the equilibrium concentration of one or both species can be obtained, for example, by NMR. This allows the calculation of the equilibrium constant, K_e_ = k_AB_/k_BA_ (as equal to C_B_(STE)/C_A_(STE)). However, the determination of the individual rate constants is impossible if no complete kinetic traces (at least one corresponding to either A , B or A and B) are available. This issue is discussed further with relation to the kinetics of thermo-photochromes (*S_11_*).

**Figure 9:**
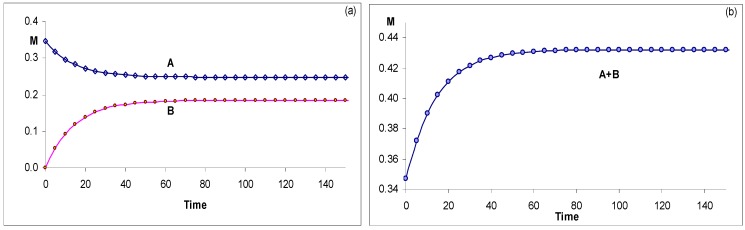
Examples of conmplete kinetic traces for AB(2k). (a) Observation of individual traces of species A and B. (b) Observation of the temporal evolution of the total absorbance of the same medium as in (a).

### Reversible Photochemical AB(2ϕ) and AB(2ϕ,1k) Systems, S_6_, S_7_ and S_8_

The two sequences S_6_ and S_7_ are presented together in this section because they are solved by the same kinetic elucidation method [94]. We consider here the typical spectral features of photochromes where the spectra of the species overlap throughout the UV region while B has an individual absorption in the visible range.

**Scheme 7:**
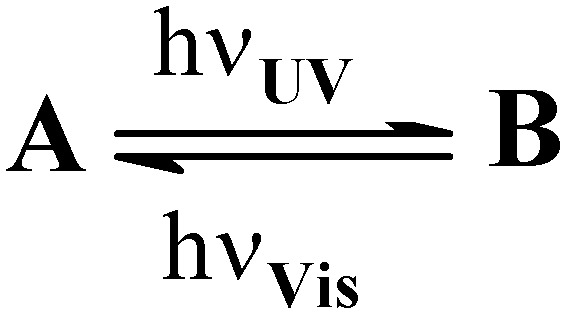
AB(2ϕ) reactions involving photoreactions activated by different lights.

There are three kinetic situations relating to the properties of the photoreactions that can be reviewed separately. The first kinetics concerns the sequence involving opposed pure photochemical reaction steps (*S_6_*) where each step is exclusively sensitive to either UV (e.g. 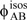) or visible light (e.g. 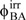, Scheme 7). The second case is that of sequences *S_6_* and *S_7_* whose photoreactions are simultaneously initiated by UV light and are independent of the irradiation wavelength. In the third kinetics we will consider the preceding case but with forward photoreaction efficiencies dependent on irradiation. The much complex kinetics where both direct and reverse quantum yields are irradiation dependent is also briefly discussed.

A simple test has been developed to check the variability of forward quantum yields with irradiation. It is given by a factor α (Eq. 22), that uses only accessible experimental data [94].

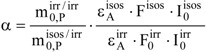
(22)
where, the value of 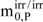 is obtained by differentiation of the non-isosbestic kinetic trace (irr/irr) and 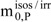 is a fitting parameter of Eq. 21a. The photokinetic factors, F^isos^ and 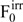, are calculated using Eqs. 3c and 3b, respectively.

For example, the quantum yields at an isosbestic (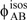) and a non-isosbestic (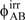) UV-irradiation wavelength are identical if α is equal to unity. Otherwise, for α ≠ 1, they are necessarily different.

### Pure photochemical opposed reactions AB(2ϕ) which are responsive to different light ranges

For this reaction, one of the species is sensitive to UV irradiation (e.g. species A) and its coloured photoproduct (e.g. species B) is only photoreactive if subjected to visible light (Scheme 7). Each reaction ends by the depletion of the irradiated species and no PSS is observed.

Since both species spectra can be recorded, the extraction of the photoreactions quantum yields can be performed by using Eq. 21a for the isosbestic UV-irradiation and Eq. 19a for the non isosbestic irradiations in the visible. It is worth noting that Eq. 19a can be rearranged to be applicable when the reaction medium is subjected to visible light (where only B absorbs) and monitored at a wavelength where both species absorb (i.e. in the UV range).

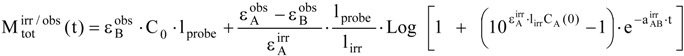
(23)

Even though such a mechanism has been proposed for photochromes [84], it is nonetheless reasonable to question the assumption employed here. Thus, unless tangible evidence is provided, the UV irradiation cannot be supposed to initiate selectively the photoreaction of species A. It is then necessary to take into account the reactivity of both species under UV-irradiation. These cases will be considered in the following sections.

### AB(2ϕ) and AB(2ϕ,1k) systems where both photochemical reactions are responsive to the same excitation beam and their quantum yields are independent of the irradiation wavelength (e.g. UV).

The pioneering work of Fischer [8] on the kinetic elucidation of AB(2ϕ) systems (*S_6_*) has demonstrated that the kinetic solution for this case can only be achieved provided that the ratio of the forward to reverse quantum yields, at any two non-isosbestic irradiations, is constant. This constant ratio hypothesis is a reasonable assumption according to Kasha’s rule. However, the mathematical formalism adopted for this method does not hold if an additional thermal reaction step is incorporated in the reaction scheme (*S_7_*). The new approach presented hereafter overcomes this problem and elucidates equally *S_6_* and *S_7_* kinetics.

The working assumption for this kinetic case supposes the invariability of the quantum yield values with the irradiation wavelength (α = 1, Eq. 22).

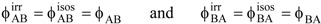
(24a)

**Figure 10:**
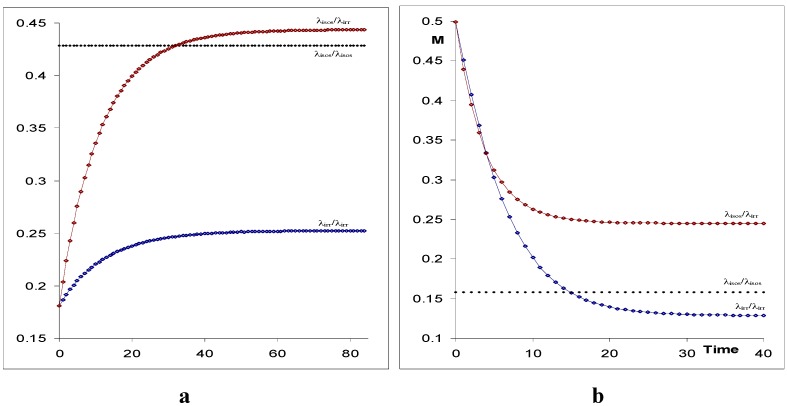
Examples of simulated (a) AB(1k,2ϕ) kinetic traces using RK integration for the photochemical reaction performed at two excitation conditions (here at exc/irr wavelength, 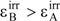) and (b) AB(2 ϕ) photoreaction (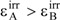 at the observation wavelength) [reprinted from Ref. 94 with permission of Photochemical and Photobiological Sciences].

The unknowns of each kinetic system are given in Table 2. The elucidation method relies on an isosbestic and a non-isosbestic irradiations in the UV-range. The corresponding kinetic traces, obtained by observing the system at a unique wavelength λ_irr_ (Figure 10), are used for the treatments.

The elucidation procedure for this kinetic cases starts with the determination of the thermal rate constant (k_BA_). It is a fitting parameter (–a_19T_) of Eq. 20b to the thermal relaxation trace. The mathematical formalism yields the analytical expression of the extinction coefficient of species B at the irradiation wavelength. The formula of 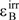 is exclusively defined by experimentally available coefficients.

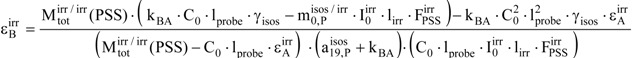
(24b)

The previous information (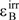, Eq. 24b) allows the determination of the absolute value of the forward quantum yield,

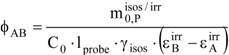
(24c)
which, in turn, serves the determination of the reverse quantum yield.

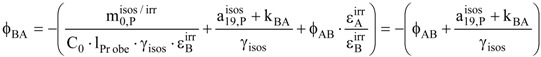
(24d)

The reconstruction of the whole spectrum of species B (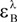) is realised through the calculation of PSS concentrations of the species as given by Eqs. 18c and 18d. These values can then be considered in the expression of the observed total absorbance (Eq.4 at PSS, i.e. 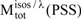 or 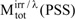) to extract the extinction coefficient values (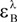).

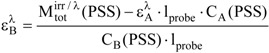
(24e)

**Table 3:** Initial parameters^a^ experimentally accessible data and fitting results for Fig 10a,b.

**Initial parameters**						
**Figure #**	C_o_(M)	(M^-1^ cm^-1^)	(M^-1^ cm^-1^)	(Einst.s^-1^ dm^-1^)	(Einstein.s^-1^ dm^-1^)	k_BA_ (s^-1^)
**10a**	1.9 10^-5^	22558	9536	2 10^-6^	9.1 10^-7^	0.04
**10b**	8.5 10^-6^	18617	58690	6 10^-6^	2.0 10^-6^	0
**Fitting results for the simulated kinetic traces and all experimentally accessible data**						
**Figure #**	(0) ^b^	^c^	^c^	γ_isos_^c^	(PSS)	
**10a**	0.1812	0.0234	-0.0889	0.0660	0.2527	1.75
**10b**	0.4988	-0.0673	-0.2651	0.2150	0.1285	1.99

^a^ Throughout l_irr_ = l_probe_ = 1 cm. ^b^: 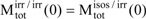. ^c^: These parameters are given in s^-1^ [reprinted from Ref. 94 with permission of Photochemical and Photobiological Sciences].

**Table 4:** Spectroscopic and kinetic values of the unknown parameters calculated by the spectrokinetic method (Eqs. 24) using data of Table 1.

**Figure #**		ϕ_AB_	ϕ_BA_	C_A_(PSS)	
				λ_isos_^a^	λ_irr_^a^
**1**	55000	0.41	0.33	1.32 10^-5^	1.74 10^-5^
**2**	10359	0.76	0.47	3.25 10^-6^	8.36 10^-7^

^a^: wavelength of the irradiation beam responsible for yielding C_A_(PSS) in solution [reprinted from Ref. 94 with permission of Photochemical and Photobiological Sciences].

The above procedure leads to the true kinetic solution for the cases targeted, for which the independence of the quantum yields from irradiation is known with certainty. If however, for a lack of convincing information, there is doubt, as it happens for a majority of new photochromes and other AB kinetic systems, then the application of the above method may lead to parameter values which have no consistent physical meaning (e.g. a quantum yield value higher than unity or negative). For these cases, the working hypothesis is invalidated; meaning that at least one of the quantum yields is irradiation dependent. Therefore, it is necessary to consider the variability of the quantum yields with irradiation wavelengths.

### AB(2ϕ) and AB(2ϕ,1k) systems where both photochemical reactions are responsive to the same excitation beam and their forward quantum yields are dependent on the irradiation wavelength.

The same experimental conditions and a set of two kinetic traces, as described in the preceding spectrokinetic method, are used for the elucidation of this dynamics [94]. For the present case we consider that ϕ_AB_ is irradiation dependent while ϕ_BA_ is constant with irradiation wavelength (Eq. 25a), i.e. α ≠ 1 (Eq. 22).

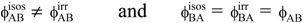
(25a)

The formalism of this kinetic elucidation case yields a second order algebraic equation for the determination of 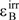 as the first unknown parameter.

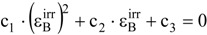
(25b)
where the constants c_i_ take the formulae below.

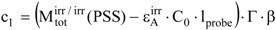
(25c)

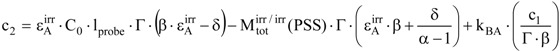
(25d)

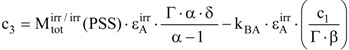
(25e)
with

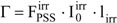
(25f)

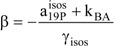
(25g)

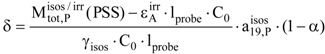
(25h)

The solution of Eq. 25b will depend on the value of the quantity, 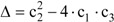; there are three possibilities available.

A negative value for Δ means that in the actual conditions there is no possible solution to the problem considered under the working assumption (see next section). However, for positive Δ and/or when Δ is equal to zero, the roots of Eq. 25b are given by,

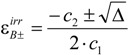
(26a)

Once the value of the extinction coefficient is obtained, the successive use of the following expressions allows the determination of the remaining unknown parameters and the completion of the kinetic elucidation of this case.

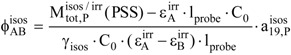
(26b)

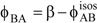
(26c)

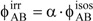
(26d)

The set of Eqs. 26 represents the kinetic solution of the systems under consideration. For those situations where a solution exists, the complete spectrum of species B is recovered in a similar manner to that proposed in the preceding method (employing Eq. 24e).

**Figure 11:**
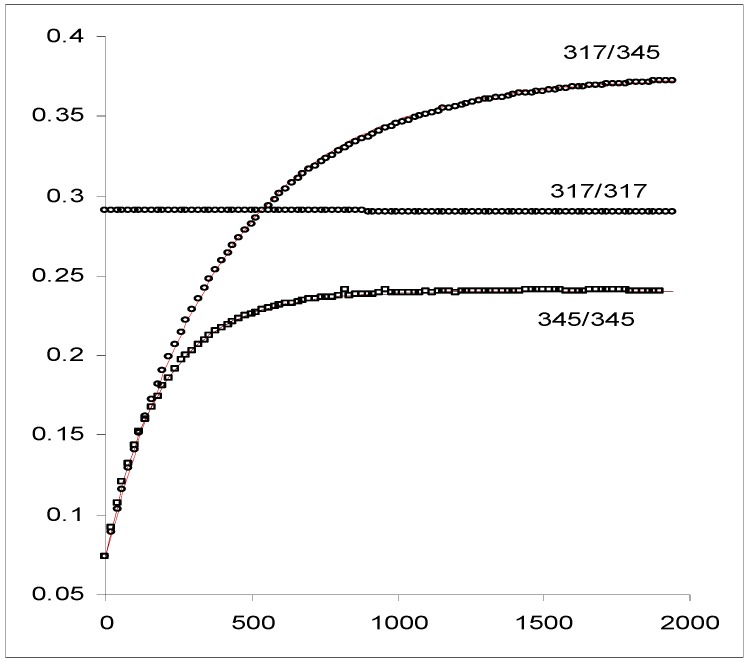
Experimental traces of DAE (5.6 10^-5^ M) in hexane at 15 °C. The kinetic traces are labelled by the wavelengths at which the irradiation and the observation were carried out (λ_exc_ / λ_obs_). Two of the curves (317/317 and 317/345, in circles) have been performed using the isosbestic irradiation and the third resulted from a non-isosbestic irradiation (345/345, in squares). The line linking the points of trace 317/345 represents the best fit to the monoexponential model, Eq.21a. The line shown on the trace 345/345 has been calculated by a RK integration using the data supplied by the elucidation method (Table 5) [reprinted from Ref. 94 with permission of Photochemical and Photobiological Sciences].

**Scheme 8:**
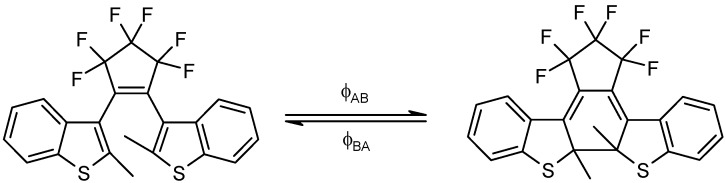
Open and closed forms of DAE.

The application of this elucidation method to the kinetic data of a diarylethene derivative, DAE, (Scheme 8) irradiated in the UV range at λ_isos_ = 317 nm and λ_irr_ = 345 nm (Figure 11), yielded the kinetic solution given in Table 5.

It is worth noting that the application of the methods developed for the kinetics of *S_3_*, and, *S_6_* and *S_7_* with ϕ ≠ f(λ_exc_), do not yield consistent results indicating that the system under consideration does not obey AB(1ϕ,1k) or AB(2ϕ,1k) kinetics with irradiation independent quantum yields. Furthermore, the use of the second root obtained from Eq. 26a (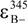 = 2094 M^-1^ cm^-1^, since Δ > 0, 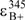 is given in Table 5 as 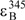) in Eqs. 26b-26d generates a negative value for ϕ_BA_ and values over unity for 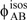 and 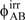.

**Table 5:** Calculated and experimental spectroscopic and kinetic values for the unknown parameters of the system.

Parameters	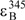 ^a^	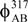	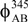	ϕ_BA_	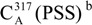 ^b^	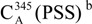 ^b^	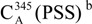 ^b^
Results of the Elucidation Method	8887.8	0.23	0.419	0.095	1.6 10^-5^	3.4 10^-5^

^a^: units: mol^-1^·L.cm^-1^. ^b^: The concentration of the initial species at the PSS (in mol·L^-1^); where the irradiation wavelength used for the experiment is also indicated.

Such results prove that the identifiability/distinguishability problem can be readily solved for this kinetics. In general terms it is crucial to review the different possibilities that can emerge from the signs and values of the roots (Δ ≥ 0) in relation to the identifiability and distinguishability issues.

In the case where the spectrokinetic method yields a unique solution, i.e. a single positive root that generates plausible results for the remaining parameters, the latter will represent the true solution for the studied kinetics since there are no identifiability or distinguishability problems. However, if both extinction coefficient values are positive (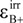 and 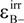 > 0), and if both lead to plausible sets ofparameter values then the identifiability and perhaps also (if ϕ_BA_ is zero for one solution) the distinguishability problems arise because of the occurrence of two options. In this case, the complete solution would require further information to discern the true solution.

### AB(2ϕ) and AB(2ϕ,1k) systems where both photochemical reactions are responsive to the same excitation beam and both their forward and reverse quantum yields are dependent on the irradiation wavelength.

The most inclusive kinetic case for AB(2ϕ,1k) systems is the one where both quantum yields are irradiation dependent, i.e. 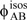 ≠ 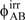 and 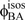 ≠ 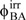. For this case, where 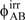 = f(λ_exc_) and 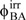 = g(λ_exc_), an analytical (assumption-free) method based on the use of pure kinetic data is yet to be developed. The difficulty in finding such a useful kinetic elucidation method stems from the fact that the number of unknowns is higher than that of the linearly independent equations that describe the reactive system. Progress on a better physical description of the kinetics by the integration of the fundamental differential equation of such kinetics would certainly represent a hope to complete this kinetic elucidation story.

Nonetheless, we ought to consider the situation where physical information is available for the system. Indeed, for AB(2ϕ) kinetics it is possible to determine the PSS concentrations of the species (e.g. by HPLC) since reactant and product are both thermally stable. The kinetic elucidation in this case is rather straightforward and requires a single trace obtained either under an isosbestic or a non-isosbestic irradiation.

The spectrum of species B is determined, in a first elucidation step, by using Eq. 24e, its PSS concentration, the spectrum of the initial species and that of the medium recorded at PSS. The forward quantum yield value can subsequently be determined in various ways, for instance, from the equation of the initial velocity; either using Eq. 27a or 27b for an isosbetic and a non-isobestic irradiation, respectively. The numerical value of the latter is obtained by differentiation of the corresponding kinetic trace as indicated above (Eq. 6).

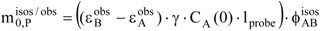
(27a)

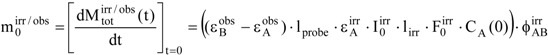
(27b)

Finally, the value of the reverse quantum yield can, for example, be extracted from the expression of C_A_(PSS) (Eqs. 8 and 17c, respectively for non-isosbestic and isosbestic irradiations).

This procedure may be repeated with data corresponding to a set of irradiations to determine the absolute values of both quantum yields throughout the UV-range. It can be combined with the application of Eq. 19a to achieve a thorough study, by determining the irradiation-dependence of species B quantum yield in the visible range.

### AB(2ϕ,1k) systems, S_8_

The spectrum of A is supposed to be inaccessible to the experimentalist, in this case. Hence, once it has completely transformed into B the kinetics can be treated as for that of S_7_ and the corresponding elucidation method applies.

### Thermophotochromic AB (1ϕ,2k) systems, S_9_ and S_10_

The kinetics of *S_8_* and *S_9_* are characterised by opposing thermal reaction steps and a single photochemical step responsible for the transformation of either A into B (*S_8_*) or B into A (*S_9_*). The total number and the identity of the five unknown parameters is the same for both kinetics (Table 2) except that each reaction sequence involves a different quantum yield (ϕ_BA_replaces ϕ_AB_ for *S_9_*). Since the lifetimes of the thermal reactions are relatively short for photochromes, recording individual spectra of initial species or the early stages of the kinetic traces by spectrophotometry is not possible. Therefore, for such kinetics the photochemical reaction is realised after the thermal equilibrium (STE) has been reached. In these circumstances, the two reaction sequences do not differ on principle in such a way that the kinetic elucidation method developed for *S_8_* [95] hereafter can similarly be used for the treatment of *S_9_* provided that the appropriate modifications due to the new reaction sequence are taken into account in the labelling of the equations.

The full elucidation of these kinetics is possible using pure kinetic data only and the formalism shows that an optimal number of one photochemical and one thermal traces is sufficient to identify all five unknowns including the spectrum of the initial species.

**Figure 12:**
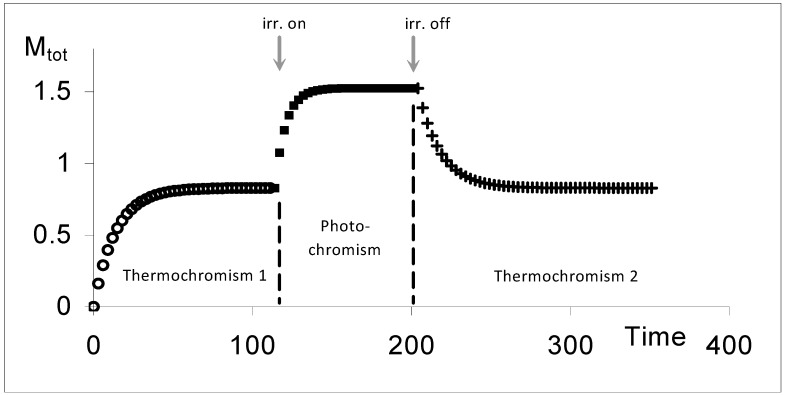
Typical kinetic traces depicting the three successive phases (Th1 (ο), P (▪) and Th2 (+)) of a thermophoto-reactive system (with M_tot_ the total absorbance of the medium at the observation wavelength; irr. on and irr. off represent respectively the start and end of the isosbestic irradiation) [reprinted from Ref. 95 with permission of the International Journal of Chemical Kinetics].

The kinetic behaviour of AB(1ϕ,2k) systems involves in theory three traces (Figure 12). The first one, is due to the thermal transformation of the initial species until the reaction reaches the state of thermal equilibrium. This stage is labelled Thermochromism 1 (or shortly Th_1_). As previously stated, this first trace in generally not experimentally accessible and its specific equations are not considered in the elucidation formalism. The second kinetic trace starting from STE, corresponds to the photochemical transformation due to an isosbestic irradiation (labelled P for photochromism). When light is switched off, the system relaxes thermally from PSS to STE during a last stage called thermochromism 2 (Th_2_).

The kinetic laws obeyed by the useful thermal and the photochemical reactions (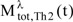 and 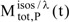 respectively), are:

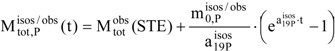
(28a)

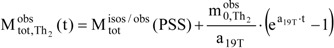
(28b)

The fitting results relative to the overall reactions’ rates are used to determine the absolute value of the quantum yield (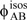) by using Eq. 21b. The knowledge of this value facilitates the calculation of the value of the molar extinction coefficient of species B at a given observation wavelength (λ).

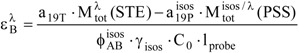
(28c)

This expression proves that it is possible to determine the spectrum of the coloured species at any observation wavelength prior to knowledge of the spectrum of the starting material and the thermal rate constants. It also shows that a previous conclusion relative to these kinetic cases relating to the necessity of knowing C_B_(STE) in order to determine the value of 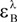 [2,3], is not valid.

In order to determine k_AB_, the absorbance of species B at the thermal equilibrium (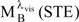) is used. (Note that in the visible region, 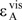 = 0).

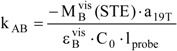
(28d)

And from there, the value of the reverse thermal rate constant is easily worked out as

k_BA_ = −a_19T_ − k_AB_ (28e)

Since all three kinetic parameter values have been defined, the four equilibrium concentrations are obtained by the application of Eqs. 17.

Finally, the kinetic elucidation is completed by deriving the extinction coefficients values at different wavelengths for species A; using the spectrum of B, the concentrations at an equilibrium state (either STE or PSS) and the full spectrum reached at that equilibrium state, as follows:

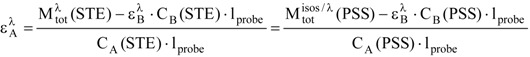
(28f)

### Thermophotochromic AB (2ϕ, 2k) systems, S_11_

The kinetics represented by a thermal and a photochemical equilibrium has not been solved by a mathematically analytical method. The solution was not achieved thus far irrespective of the data used (i.e., using only kinetic or a mixted set of kinetic and physical data).

The reason that hampers devising a mathematically analytical elucidation remains, here as well, an excessive number of unknowns compared to that of the available equations. The serious identifiability problem, which is inherent to this kinetic elucidation, is unfortunately unsolvable.

However, the thermal rate constants can be obtained if kinetic and physical information are combined. For example, let the concentration of the species at STE as predicted for AB(2k) systems (*S_5_*), be obtained by a physical method (e.g. NMR, realised at the same temperature and the same solvent as that of the kinetic study). The kinetic data required are represented by a set of one thermal and one photochemical trace (realised under an isosbestic irradiation). In these conditions, the system will formally behave similarly to Figure 12.

The kinetic traces are fitted with Eqs. 28a and 28b, and their respective fitting coefficients defined. According to Eqs. 17a and 17b, the ratio of STE species concentrations is equal to the ratio of the rate constants, as:

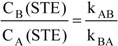
(29a)

Combining Eq.29a and the fitting coefficient a_19T_ (Eq.14b) will allow the extraction of the absolute values for the individual rate constants of the thermal reactions (k_AB_ and k_BA_). Also, the value of the sum of quantum yields is obtained as follows:

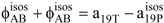
(29b)

For this case, unfortunately, the remaining individual values of quantum yields and extinction coefficients for both species cannot be obtained analytically.

## 10. Determination of reaction quantum yields at any irradiation wavelength

The knowledge of the possible variability of the quantum yield with irradiation wavelengths is important for both application and theoretical purposes. The methods proposed above for the elucidation of AB kinetics provide the absolute values for the quantum yields at specific wavelengths (generally either at λ_isos_ or at both λ_isos_ and λ_irr_). This is true for the solved kinetics involving photoreactions (*S_1_*, *S_3_*, *S_4_*, *S_6_*-*S_10_*). 

Since the kinetic laws for non-isosbestic irradiations are not known with the exception of *S_1_* (Eq. 19), the determination of the values of the quantum yields at different irradiations must be carried out after the kinetic elucidation is completed in the conditions set in Section 9. In these circumstances, the spectra of both species as well as the thermal rate constants are all available. This is important because if the reaction, subjected to the new non-isosbestic irradiation wavelength (labelled irr_x_), is performed with all experimental conditions similar to those that served for the kinetic elucidation (of Section 9) then the photochemical quantum yields values are the only unknowns of the new kinetics. Accordingly, the absolute value of the forward quantum yield (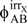) can be derived by several ways; one of which being the use of the initial velocity Eq. 6, as:

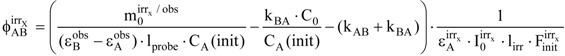
(30)

For reaction sequences involving a second photochemical step (*S_6_*–*S_8_* and *S_10_*), the value of the reverse quantum yield (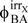) can be worked out from the equation of the total absorbance at the PSS (Eq. 9). Hence, individual dependence of the efficiencies (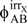 and 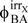) on the irradiation wavelength can easily be studied.

## 11. Applicability of the kinetic elucidation methods to non-chromic systems

Those methods described in Section 9 that do not require in their elucidation procedure data relative to a chromic species (e.g. species B absorbing alone in the visible) are applicable to the elucidation of the kinetics of non-chromic systems.

## 12. Conclusions

The paper reviews all possibilities of kinetic elucidations relating to AB systems. Simple and easy-to-implement procedures are provided. The elucidation methods presented in this paper tackle and in most cases solve the complex identifiability/distinguishability problem. Their performance and usefulness for photochromes and non-chromic materials have also been shown through a number of examples.

## 13. Glossary

### Labelling


AB (nk,m)Bimolecular kinetic systemASpecies A (initial reactant)BSpecies B (thermo- or photoproduct)ABTransformation of species A into BBATransformation of species B into AλAn arbitrary wavelengthobsObservation wavelength (_obs_)irrIrradiation wavelength (_irr_)isosIsosbestic-point wavelength (_isos_)0Index or argument corresponding to time zero of the reactionPSSIndex or argument relative to the reaction at the Photo-Stationary StateSTEIndex or argument relative to the reaction at the State of thermal EquilibriuminitIndex or argument equal to 0, STE or PSSPPhotochemical processTPurely thermal processΩIndex standing for either P or T lettersf(λ_exc_)A function dependent on the excitation wavelengthg(λ_exc_)A function dependent on the excitation wavelength


### Irradiation and observation conditions


λ_exc_An arbitrary wavelength used to irradiate the sample; it includes _isos_ which is the wavelength of an isosbestic point and _irr_ (≠ _isos_) an irradiation wavelength where (and both ≠ 0).λ_obs_An arbitrary observation wavelength (which might be equal to _irr_)
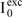
Incident light intensity of the excitation beam at λ_ιρρ_(${\text{I}}_{0}^{\text{irr}}$) or _isos_ (${\text{I}}_{0}^{\text{isos}}$)l_probe_Optical path length of the spectrophotometer (probing) light inside the samplel_irr_Optical path length of the irradiation light inside the sample


### Concentrations


C_i_(t)Concentration of species *i* = A or B at time tC_i_(PSS)Concentration of species *i* = A or B at time PSSC_i_(STE)Concentration of species *i* = A or B at time STEC_0_Total concentration of the species in the reactive medium


### Kinetic parameters



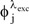
Generic quantum yield of the transformation *j* when the reaction medium is subjected to an excitation beam whose wavelength is λ_εξχ_ (= λ_ισοσ_ or λ_irr_).
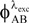
Quantum yield of the transformation of A into B at the excitation wavelength λ_exc_
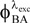
Quantum yield of the transformation of B into A at the excitation wavelength λ_exc_k_BA_First-order rate constant of the thermal transformation of B into A or A into BF^irr^(t)Photokinetic factor at the irradiation wavelength and time tF^isos^(or F^isos^(t) = F^isos^) Constant photokinetic factor at λ_isos_
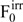
(or F^irr^(0)) Constant photokinetic factor at λ_irr_ and t = 0
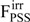
(or F^irr^(PSS)) Constant photokinetic factor at λ_irr_ at PSS
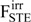
(or F^irr^(STE)) Constant photokinetic factor at λ_irr_ at STE
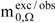
Initial velocity of reaction Ω when irradiation has been carried out using λ_exc_ (= isos or irr) and observed at λ_obs_ (= irr)a_19Ω_Overall first-order reaction rate for the process Ω (λ_exc_ = λ_isos_)a_4Ω_First-order direct reaction rate for the process Ω (λ_exc_ = λ_isos_)a_5Ω_First-order reverse reaction rate for the process Ω (λ_exc_ = λ_isos_)γ_isos_Constant factor that multiplies quantum yields when λ_exc_ = λ_isos_


### Spectroscopic parameters



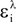
Extinction coefficient of species *i* (A or B) at λ (= isos, irr or obs)
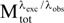
The total absorbance of the medium measured at various excitation/observation wavelengths’ combinations (e.g. 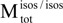, 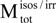 and 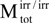 respectively at isos/isos, isos/irr and irr/irr)
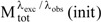
Particular constant values of the total absorbance measured at init = 0, PSS, STE


### Acronyms


HPLCHigh Performance ChromatographyMCMerocyanine moleculeNMRNuclear Magnetic ResonanceRKRunge-Kutta numerical integration methodSPSpiropyran moleculeTTCTrans-Trans-Cis isomer of spiropyran (the same meaning for T and C applies for TTT, CTT and CTC)UVUltraviolet spectrophotometry or rangeV_is_Spectrophotometry or spectral range relating to the visible range of the electromagnetic spectrum

